# Immediate versus delayed implant placement in the esthetic zone: a prospective 3D volumetric assessment of peri-implant tissue stability

**DOI:** 10.1186/s40729-022-00457-9

**Published:** 2022-11-25

**Authors:** Puria Parvini, Katharina Melissa Müller, Emilio A. Cafferata, Frank Schwarz, Karina Obreja

**Affiliations:** 1 Department of Oral Surgery and Implantology, Goethe University, Carolinum, Frankfurt Am Main, Germany; 2 Department of Periodontology, School of Dentistry, Universidad Científica del Sur, Lima, Peru

**Keywords:** Immediate implantation, Delayed implantation, Intraoral scan, Volumetric analysis

## Abstract

**Purpose:**

To evaluate the volumetric stability of peri-implant soft and hard tissue prospectively, this study compared immediate versus delayed implants placed in the anterior esthetic region.

**Methods:**

This non-randomized controlled clinical study included 25 patients, who received an immediate (type 1) or a delayed (type 4) implant placement for the replacement of a single anterior tooth. The anterior maxillae were intraorally scanned at three timepoints: before surgery (S0), 6 months (S1), and 12 months (S2) after surgery. A specific region of interest (ROI), divided into marginal and apical regions, was determined and superimposed for volumetric changes analysis. At 6 and 12 months, the probing depth (PD), bleeding/suppuration on probing (BOP/SUP), modified plaque index (PI), keratinized mucosa (KM) width, mucosal recession (MR), and implant stability (PTV) by means of periotest were recorded.

**Results:**

Between S0–S2, tissue surrounding immediate implants was reduced in 0.37 ± 0.31 mm, whereas delayed implants gained 0.84 ± 0.57 mm mean tissue volume. Peri-implant tissue loss at type 1 implants occurred primarily in the marginal section of the ROI (0.42 ± 0.31 mm), whereas tissue gain at type 4 implants occurred mainly in the apical section (0.83 ± 0.51 mm). These values were significantly different between both groups for the entire ROI (*p* = 0.0452) and the marginal region (*p* = 0.0274). In addition, the mean buccal KM width around type 1 implants was significantly wider in comparison with the type 4 implants group after 12 months (*p* = 0.046). There were no significant differences between groups regarding PD, BOP/SUP, or PTV.

**Conclusions:**

The results suggest that type 1 implants placed in the esthetic region experience more tissue loss than type 4 implants, thus marginal tissue remodeling should be considered for planning immediate implants placement in the anterior maxillae.

**Graphical Abstract:**

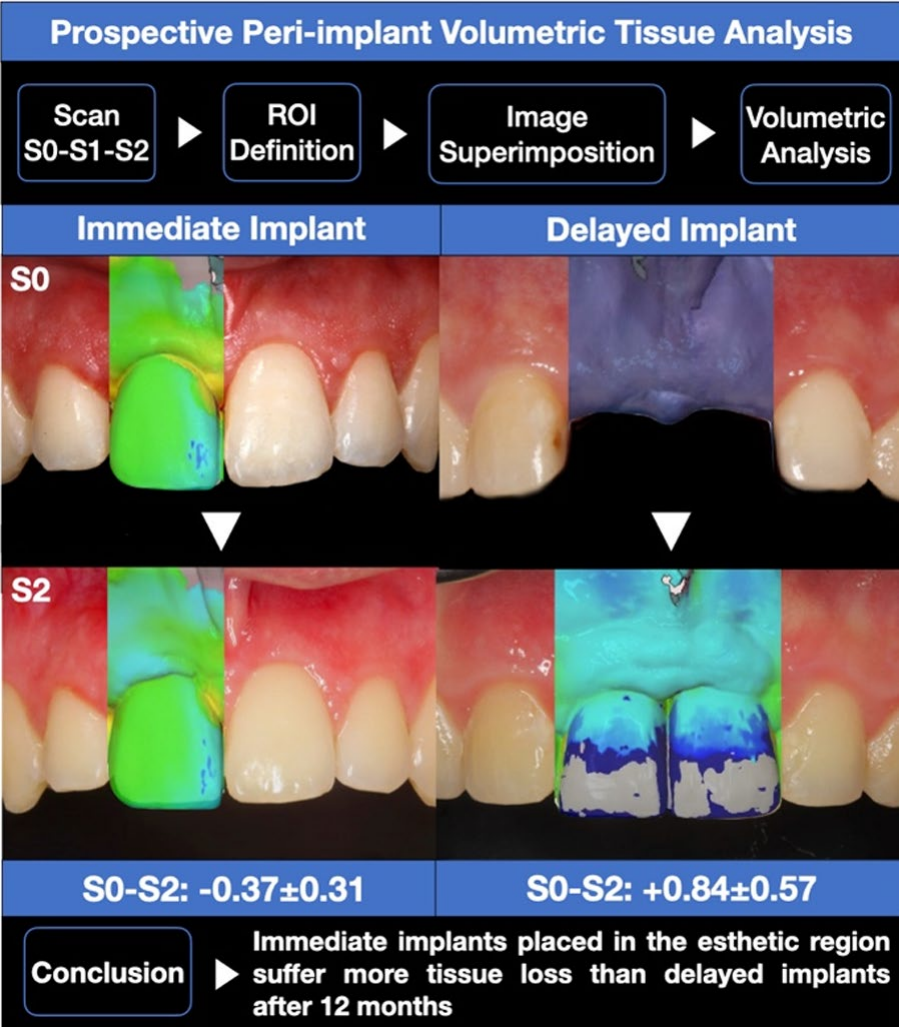

## Background

Prosthetic replacement of unrestorable anterior teeth is still one of the major clinical challenges for dentists. Implant-supported restorations offer a highly reliable and extensively documented treatment option [1]. However, implantation time decision making can affect the esthetic prognosis of the final restoration [2]. In this sense, delayed implant placement protocols present high implant/restoration survival rates and stable esthetic outcomes but require additional surgical intervention and substantial healing time until final prosthesis deliver. Alternatively, immediate implant placement protocols provide similar implant/restoration survival rates but with higher patient satisfaction [3, 4].

Immediate implantation and loading can be performed in select cases, allowing for tooth extraction and implant placement in the same session, and consequently leading to reduced patient treatment time and morbidity [5, 6]. For this, a thick gingival biotype, a remaining buccal bone wall, and a palatally positioned implant are needed to avoid the occurrence of marginal dehiscences that negatively affect esthetic outcomes [7]. Although an enhanced maintenance of peri-implant hard and soft tissue margins can be achieved in these cases, the incomplete preservation of facial soft tissue contours followed by physiological alveolar bone resorption often leads to suboptimal esthetic results [8].

Therefore, the importance of the assessment of peri-implant soft and hard tissue dynamics becomes mandatory for predicting long-term treatment stability [9]. In this context, emerging 3D intraoral scanning technologies offer an accurate evaluation of peri-implant tissues without exposing patients to radiation or uncomfortable impressions [10]. Moreover, time-dependent volumetric evaluations of peri-implant tissue dynamics are still scarce [10, 13]. Thus, the main objective of this study was to prospectively evaluate the 3D volumetric stability of peri-implant soft and hard tissue, comparing type 1 and type 4 implants placed in the anterior esthetic region. The primary outcome measure was peri-implant tissue volumetric changes and the secondary outcome measures were peri-implant health by means of probing depth and bleeding/suppuration on probing, modified plaque index, keratinized mucosa width, mucosal recession, and implant stability by means of periotest. Being the null hypothesis: there is no difference between immediate and delayed implant placement in the esthetic zone, regarding volumetric peri-implant tissue changes.

## Methods

### Study design and participants

This non-randomized controlled clinical study included a total of 25 patients (female/male: 3/22; age mean ± standard deviation: 58.69 ± 15.07), recruited at the Department of Oral Surgery and Implantology, Goethe University, Frankfurt, being in need for at least one implant-supported single-tooth restoration in the esthetic zone (i.e., anterior maxilla including teeth 15–25). The patients were candidates for either an immediate implant placement (i.e., type 1) in the presence of a non-retainable tooth (*n* = 16 implant sites) or a late implant placement (> 4 months after tooth extraction) (i.e., type 4) in the presence of a single-tooth tooth gap (*n* = 16 implant sites) [7].

Each patient was given a detailed description of the study procedures and signed an informed consent before participation. The study was conducted in accordance with the Helsinki Declaration, as revised in 2013, and was approved by the local ethics committee (N: 19-233). The present study considered the STROBE statement checklist items for its reporting [11].

### Inclusion and exclusion criteria

The inclusion criteria for patient selection were the following:

- Patient age ≥ 18 years.
- Mental and physical ability to comprehend the aim, risks, and benefits of the study, and willingness to reliably attend the follow-up appointments.

The exclusion criteria were:

- Uncontrolled systemic diseases that could affect bone remodeling during implant osseointegration or soft tissue healing (e.g., uncontrolled diabetes mellitus [HbA1c > 7], osteoporosis).
- Current use of medications that could affect bone remodeling during implant osseointegration or soft tissue healing (e.g., steroids, antiresorptive medication, radiotherapy).
- Pregnancy or lactation periods.

### Surgery and prosthetic protocol treatment procedures

Prior to surgery, all patients were prescribed amoxicillin 1000 mg to be taken three times daily for 7 days, starting 1 day before the surgical intervention. Moreover, patients were instructed to rinse with a 0.2% chlorhexidine mouthwash for 1 min.

All surgical interventions were performed under local anesthesia (Ultracain D-S 1:200.000, Sanofi-Aventis, Germany) by the same experienced oral surgeon (P.P.). In the case of type 1 implant placement, special care was taken to perform a flapless atraumatic tooth extraction. If a root remnant had to be extracted, the Benex system (Helmuth Zepf Medizintechnik GmbH, Seitingen-Oberflacht, Germany) was used. Next, granulation tissue was removed, and the alveolar socket was irrigated with sterile saline, while the integrity of the buccal bone wall was evaluated. Then, the implant site was prepared following the manufacturer’s indications, and the implant was free hand placed directly facing the palatal bone wall in a subcrestally ideal prosthetic position. In the case of type 4 implant placement, a minimally invasive full thickness flap was raised at the edentulous site, followed by a conventional drilling protocol as recommended by the manufacturer. Afterward, the implant was placed subcrestally in an ideal prosthetic position. Whenever bone augmentation was considered necessary, a bovine-derived bone substitute (Geistlich BioOss; Geistlich-Pharma, Wolhunsen, Schwitzerland) mixed with autogenous bone chips, obtained from the implant site preparation, was used for gap filling, or in combination with a resorbable collagen membrane (Geistlich BioGide; Geistlich-Pharma, Wolhusen, Schwitzerland) in the cases presenting buccal bone dehiscences for lateral augmentation. Surgical sites were closed with monofilament PTFE sutures (Cytoplast 5–0; Daikin America Inc.; New York; USA) and a postoperative panoramic radiograph was taken. Patients were instructed to rinse with a 0.2% chlorhexidine mouthwash two times a day for 1 week.

Finally, a temporary or definitive abutment (“One Abutment-One Time”) was placed, accompanied by a non-occlusive temporary crown [12]. Whenever the “One Abutment-One Time” was used, a Scanbody was inserted to detect the implant position by performing an intraoral Scan (3 Shape TRIOS MOVE, Germany). Next, an individually manufactured/milled abutment and provisional crown were fabricated by CAD/CAM and delivered the same day.

Sutures were removed after seven and up to 14 days later. Definitive prosthetic restorations were installed after 3–6 months. Patients were included in a maintenance program and re-evaluated at 6 and 12 months.

### Data acquisition and follow-up/clinical assessment

The following clinical parameters were assessed postoperatively during the controls using a periodontal probe (PCP UNC 15 Hu-Friedy Inc., Chicago, Illinois, USA), at baseline, 6, and 12 months:

- Probing depth (PD), defined as the distance from the gingival margin to the bottom of the probing pocket.
- Bleeding and/or suppuration on probing (BOP/SUP), appearing within 30 s after probing.
- Plaque Index (PI), measured as presence or absence of plaque.
- Keratinized mucosa (KM) width, measured from the restoration margin to the mucogingival junction.
- Mucosal recession (MR) of the peri-implant soft tissue, measured from the restoration margin to the mucosal margin.
- Periotest value (PTV), considered as maximum value of implant stability.

PD, BOP/SUP, and PI were assessed at six sites per implant (mesio-buccal, mid-buccal, disto-buccal, mesio-oral, mid-oral and disto-oral) by two calibrated investigators (K.M. and K.O.).

The calibration of the investigators was performed prior to the beginning of the study, for the standardization of data collection and clinical assessment. For each clinical variable, each investigator performed a first measurement, and then repeated the measurement in the same site after 5 min. Calibration was considered successful when the measured values of the repeated measurement procedures matched > 95%. Documentation of demographic study variables, implant site characteristics/features, and clinical measurements were also recorded using a standardized documentation form.

### Digital/volumetric analysis

**Fig. 1:**
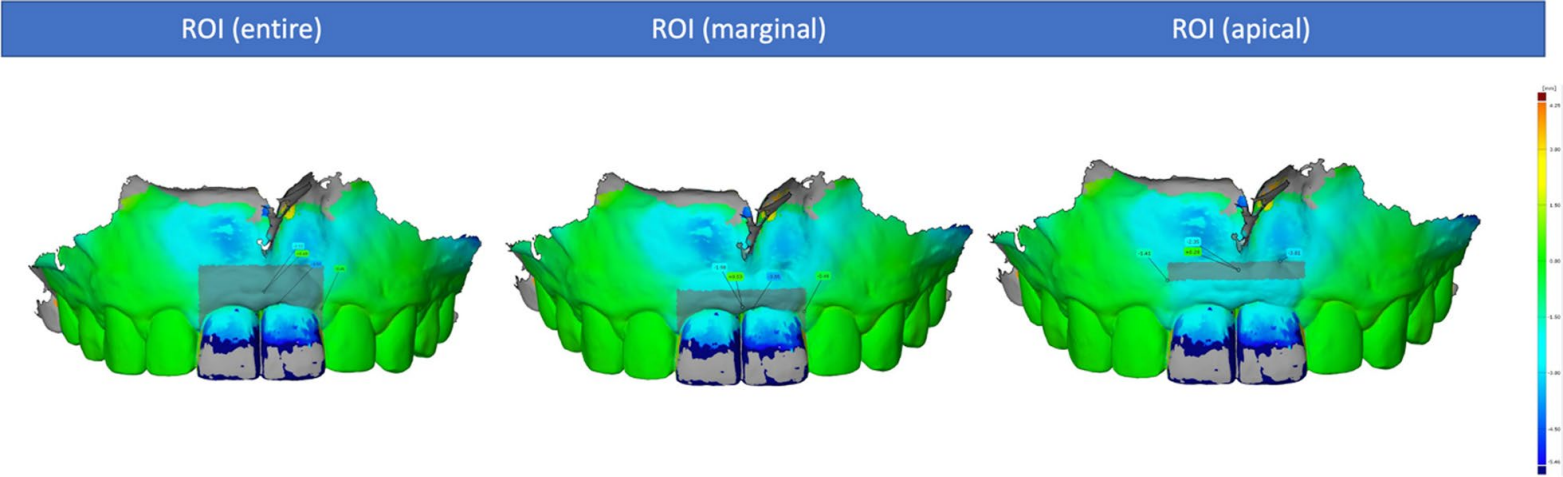
Delimitation of the region of interest (ROI)

**Fig. 2:**
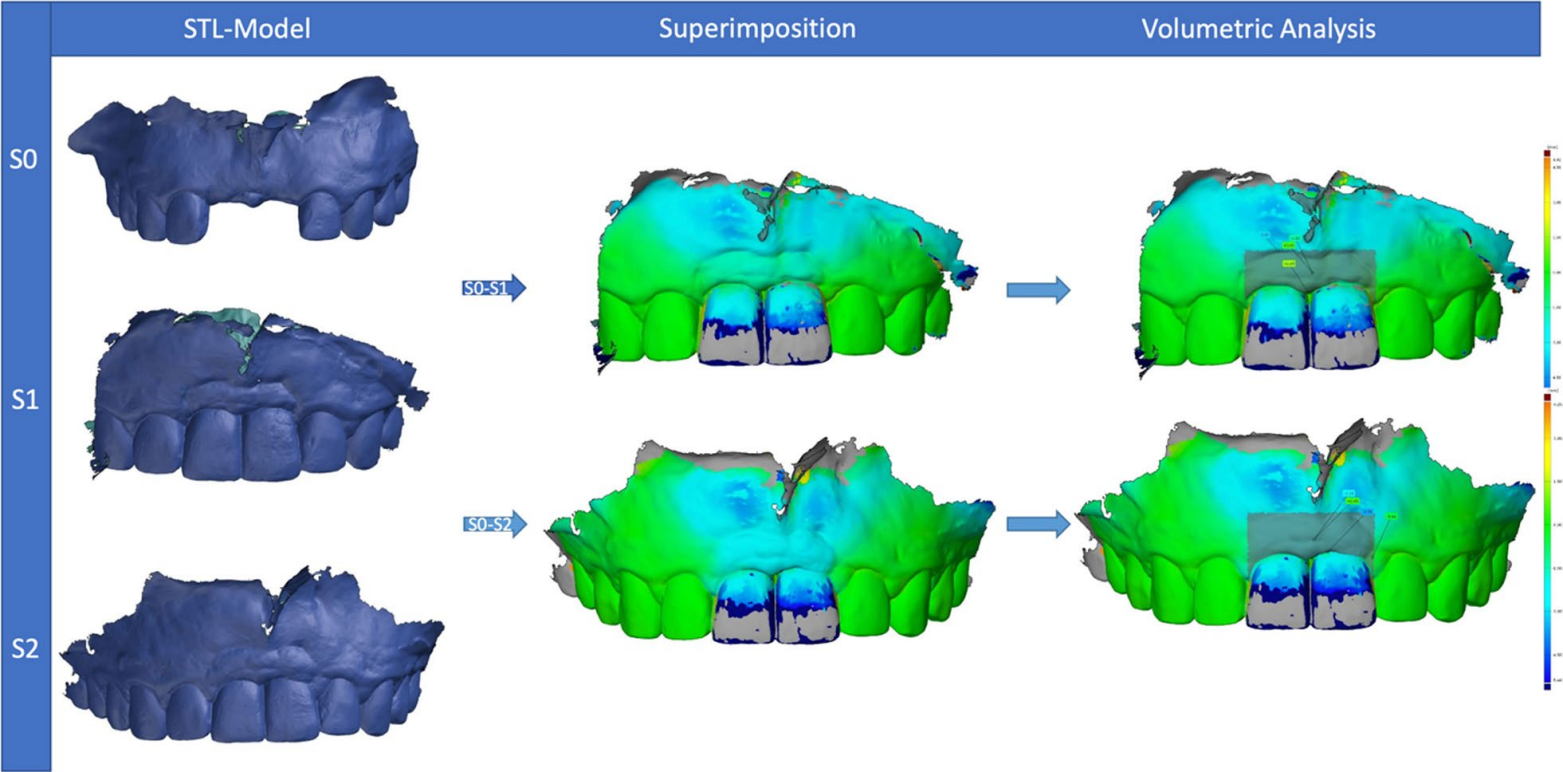
Image superimposition and volumetric analysis

Before the patients received the surgical intervention, a preoperative intraoral scan (S0) was made using an intraoral scanner (3 Shape TRIOS MOVE, Germany GmbH) to provide the baseline scan for the generation of the region of interest (ROI) and to digitally fabricate the provisional crown, in the case of immediate implant loading. Having a previously defined ROI, scans were performed at the follow-ups at 6 (S1) and 12 months (S2) after the surgery. These scans were subsequently exported as STL (standard tessellation language) files and served as a basis for the dynamic analysis of peri-implant soft tissue changes. For this, the scan files of the different timepoints were superimposed using the mesh processing software system MeshLab (Meshlab, ISTI, Italy, 2016). Then, the models were aligned on the CAD model (computer aided design) using at least 8 reproducible anatomical structures, including teeth or prosthetic crowns, and subsequently converted to STL files. These were imported into GOM software (GOM inspect Suite 2020, Zeiss Company, Braunschweig, Germany) for digital measurement and volumetric analysis. By superimposing and comparing the delimited areas within files, changes in the ROI could be quantified. This ROI was defined as follows: apically 4 mm from the highest point of the marginal gingiva, laterally to the center of the papilla of the implant crown, and coronally to the height of the marginal gingiva (Fig. 1). Furthermore, the selected ROI was divided into two equivalent sections (i.e., marginal and apical sections) to represent the areas with the most volume change. This process was applied for a comparison of the CAD models S0–S1, S0–S2, and S1–S2, to show a time-dependent course of the volumetric peri-implant tissue changes (Fig. 2).

All measurements were performed by the same experienced and calibrated examiner (K.M.). Each analysis was conducted in triplicate. Prior to the analysis, an intra-examiner calibration was performed to determine the reproducibility of the measurements. The calibration was accepted when ten repeated measurements presented an intraclass correlation coefficient ranging from 0.81 to 1.

### Statistical analysis

The clinical and the intraoral scan measurements were collected pseudonymously in a standardized sheet (Microsoft Excel Version 16.43, Microsoft Corporation, Redmond, USA). Data were expressed as means and standard deviations, whereas nominal data were presented as absolute and relative frequencies. The normal distribution of the data was assessed by the Shapiro-Wilk test. The Mann-Whitney *U* test was used for independent variables, the Wilcoxon test was used for dependent variables, and the sign test was used for the ordinal data analysis. The categorical or nominal data was assessed by applying the chi-square test or Fischer's exact test. Moreover, the comparison between dichotomized data of the different timepoints was done with the modified chi-square test according to McNemar. Apart from that, a binary logistic regression analysis was performed to investigate any association between dependent binary and independent variables. In addition, a paired *T* test was performed for the continuous data of the volumetric analysis. A two-sided test of significance was performed for all test procedures, with significance at a *p* value < 0.05. A statistical software (IBM SPSS Statistics 24.0, IBM Deutschland GmbH, Ehningen, Germany) was used to analyze the compiled data.

## Results

### Patients and implants characteristics

This study included 25 participants who received a total of 32 implants. In the type 1 implant group, 14 patients received 1 implant each and 1 patient received 2 implants (*n* = 16 implant sites). In the type 4 implant group, 11 patients received 1 implant each, 1 patient received 2 implants, and 1 patient received 3 implants (*n* = 16 implant sites). Among the participants, 3 of them received both type 1 and type 4 implants, 2 patients received 1 type 1 and 1 type 4 implant, and 1 patient received 1 type 1 and 3 type 4 implants. Therefore, type 1 and type 4 placed implants were equally distributed in both groups (16 type 1 and 16 type 4).

All the implants were placed in the anterior maxilla and were of the same self-taping bone-level type (BLX-Implant system, Straumann Holding AG, Basel, Switzerland). Most of the implants were immediately loaded, following the One Abutment–One Time concept (20 implants, 62.5%). Though, 14 patients (43,8%) had a history of periodontitis, no complications were recorded during the healing process and no implants were lost during the observation period of 12 months. In addition, 1 patient was a smoker, who presented no signs of inflammation after 6 or 12 months. Furthermore, no signs of progressive bone loss compatible with peri-implantitis were detected. Table 1 shows the implants distribution and their characteristics.

**Table 1:** Implant characteristics at the implant level (*n* = 32)

	Type 1	Type 4	Total
Implant position			
11, 21	3 (18.75%)	3 (18.75%)	6 (18.75%)
12, 22	6 (37.50%)	3 (18.75%)	9 (28.13%)
13, 23	4 (25.00%)	1 (6.25%)	5 (15.63%)
14, 24	2 (12.50%)	5 (31.25%)	7 (21.88%)
15, 25	1 (6.25%)	4 (25.00%)	5 (15.63%)
Bone grafting	15 (93.75%)	8 (50.00%)	23 (71.88%)
Gap filling	13 (81.25%)	0	13 (81.25%)
LRG	2 (12.50%)	9 (56.25%)	11 (68.75%)
OAOT	14 (87.50%)	6 (37.50%)	20 (62.50%)
Primary stability (mean ± SD; Ncm)	42.19 ± 9.12	41.25 ± 18.12	41.72 ± 14.12

*LRG* lateral ridge augmentation, *OAOT* one abutment-one time concept

### Clinical parameter analysis

The clinical parameters, PD, BOP, PI, KM width, MR, and PTV, recorded at 6 and 12 months, are shown in Table 2. There were no significant differences between type 1 and type 4 implant groups in terms of PD, BOP, MR, or PTV at the different timepoints (*p* > 0.05). Only the mean buccal KM width around type 1 implants was significantly wider in comparison with the type 4 implants group after 12 months (*p* = 0.046).

**Table 2:** Clinical parameters (mean ± SD) at 6- and 12-month follow-ups, at the implant level (*n* = 32)

Clinical parameters	Type 1 Implants 6 months	Type 4 Implants 6 months	*p* value	Type 1 Implants 12 months	Type 4 Implants 12 months	*p* value
PD (max; mm)	3.22 ± 0.84	3.38 ± 1.15	0.867	3.25 ± 0.58	3.28 ± 0.73	0.914
BOP (%)	5.21 ± 10.03	2.08 ± 5.69	0.345	7.29 ± 12.12	8,33 ± 12,17	0.753
PI (mean ± SD)	0.34 ± 0.48	0.18 ± 0.38	0.029*	0.18 ± 0.38	0.18 ± 0.38	1.000
KM width (buccal; mm)	4.94 ± 1.44	4.38 ± 1.09	0.154	5.25 ± 1.18	4.44 ± 1.03	0.046*
MR (buccal; mm)	0	0	1.000	0	0.06	0.317
PTV	0.31 ± 1.49	− 0.87 ± 0.82	0.062	0.06 ± 1.69	− 088 ± 2.47	0.536

*PD* probing depth, *BOP* bleeding on probing, *PI* plaque index, *KM* keratinized mucosa, *MR* mucosal recession, *PTV* periotest-value

*p < 0.05

No significant differences were detected regarding the PD, BOP, PI, KM width, MR, or PTV between the 6- and 12-month follow-up appointments within the groups (*p* > 0.05).

### Digital/volumetric analysis

The peri-implant tissues of both groups initially showed a mean volume gain of 0.03 ± 0.43 mm after 6 months (S0 to S1), and a total of 0.09 ± 0.41 mm after 12 months (S0–S2). After 6 months (S0–S1), the analysis showed that this change occurred mainly in the peri-implant apical region, with an initial volume gain of approximately 0.08 ± 0.32 mm. Otherwise, a loss of 0.06 ± 0.37 mm was detected within the peri-implant marginal region during the same period (S0–S1) (Table 3).

**Table 3:** Volumetric tissue changes (mean ± SD) at the 6- and 12-month follow-up

Time	ROI	Type 1 implants	Type 4 implants	*p* value	Total
S0–S1 (mm)	ROI	− 0.53 ± 0.30	0.94 ± 0.64	0.0021^*^	0.03 ± 0.43
	Marginal ROI	− 0.60 ± 0.26	0.83 ± 0.55	0.0004^*^	− 0.06 ± 0.37
	Apical ROI	− 0.31 ± 0.19	0.62 ± 0.50	0.2321	0.08 ± 0.32
S0–S2 (mm)	ROI	− 0.37 ± 0.31	0.84 ± 0.57	0.0452^*^	0.09 ± 0.41
	Marginal ROI	− 0.42 ± 0.31	0.80 ± 0.49	0.0274^*^	0.04 ± 0.38
	Apical ROI	− 0.16 ± 0.17	0.83 ± 0.51	0.1812	0.26 ± 0.31
S1–S2 (mm)	ROI	0.10 ± 0.12	0.08 ± 0.23	0.8274	0.09 ± 0.17
	Marginal ROI	0.11 ± 0.12	0.11 ± 0.20	0.9129	0.11 ± 0.16
	Apical ROI	0.07 ± 0.07	0.02 ± 0.20	0.8258	0.04 ± 0.14

*p < 0.05

After 12 months (S0–S2), a mean gain of 0.26 ± 0.31 mm was recorded within the peri-implant apical region, in which the greatest change occurred. In addition, an average gain of 0.04 ± 0.38 mm was detected in the marginal region during this time period (S0–S2).

**Fig. 3:**
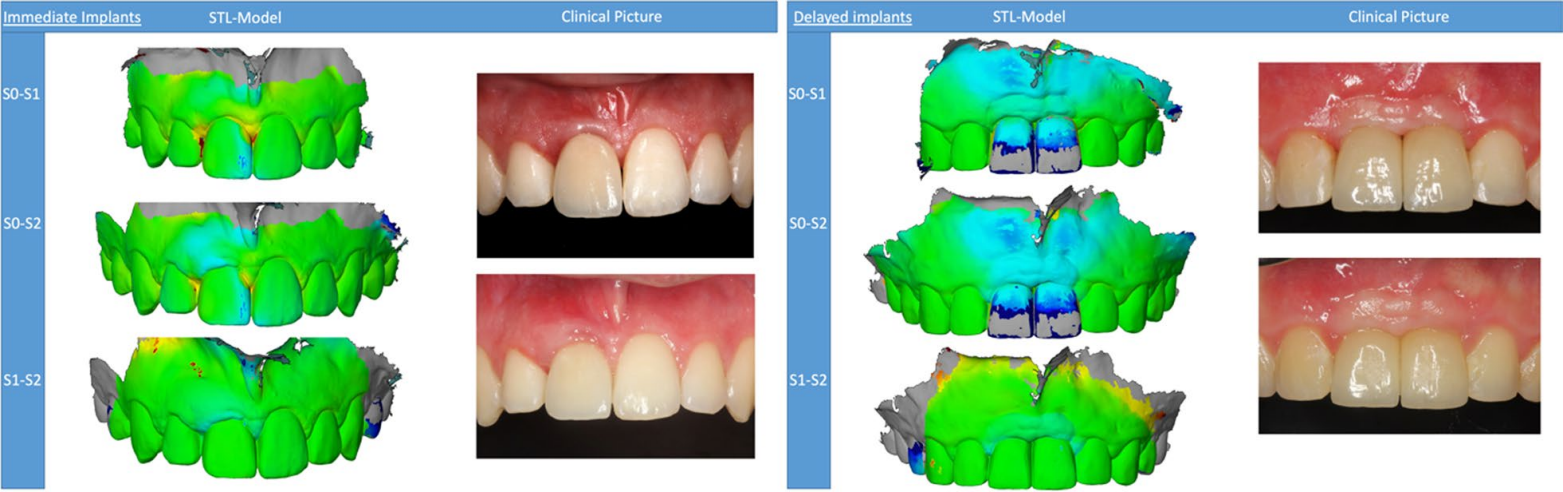
Representative volumetric superimposition and clinical images. **A** Immediate implantation group, **B** delayed implantation group

When comparing the volumetric tissue changes between type 1 and type 4 implants, the following changes were observed (Fig. 3).

During the first 6 months after implant placement (S0–S1), a mean tissue loss of 0.53 ± 0.3 mm occurred throughout the selected area of interest (ROI), mainly in the marginal region (− 0.6 ± 0.26 mm) around type 1 implants. In contrast, peri-implant tissues around type 4 implants showed a mean gain of 0.94 ± 0.64 mm during this observation time, also mainly in the marginal region (0.83 ± 0.55 mm). In comparison, there were significant differences between type 1 and type 4 peri-implant tissue volumes for the entire ROI (*p* = 0.0021), as well as for the marginal region (*p* = 0.0004).

In the period between 6 and 12 months (S1–S2), there was a mean peri-implant volume increase of 0.1 ± 0.12 mm for type 1 placed implants, and a 0.08 ± 0.23 mm volume increase for type 4 placed implants. These peri-implant tissue changes occurred mainly in the marginal region in both groups (type 1: 0.11 ± 0.12 mm; type 4: 0.11 ± 0.2 mm). In comparison, there were no significant differences between the groups (*p* > 0.05) during this time period (S1–S2).

**Fig. 4:**
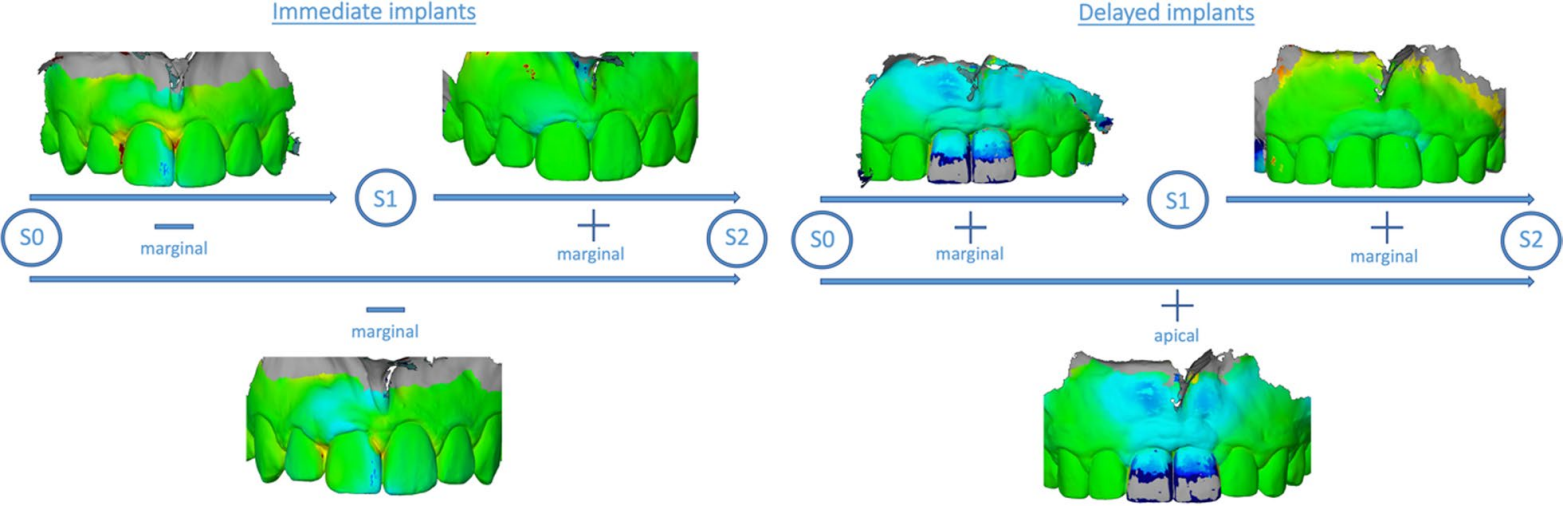
Volumetric tissue changes after 6 (S0–S1) and 12 (S0–S2) months, and comparing baseline with 12 months (S0–S2). **A** Immediate implantation group, **B** delayed implantation group

**Fig. 5:**
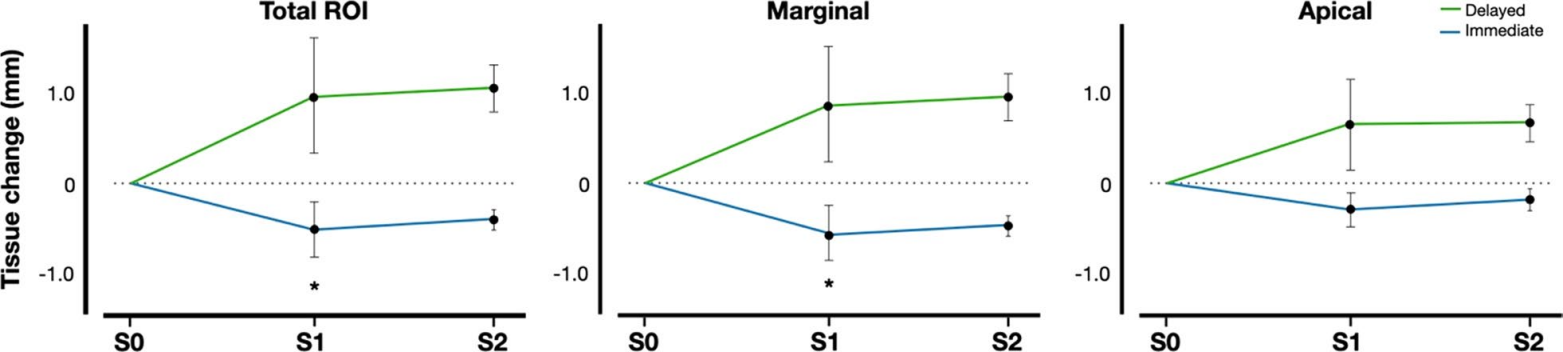
Peri-implant tissue volume changes after 6 (S0–S1) and 12 months (S1–S2) in immediate and delayed placed implants groups. **A** Entire ROI, **B** marginal, and **C** apical sections. Values are expressed as mean ± SD of 16 implants per group. *p < 0.05

When comparing the baseline and 12-month values (S0–S2), a mean volumetric loss of tissue of 0.37 ± 0.31 mm for type 1 implants was detected, whereas a mean volume gain of 0.84 ± 0.57 mm was detected in the type 4 implants group. The changes within the peri-implant tissues over the entire 12-month period around the type 1 implants primarily occurred in the marginal section of the ROI, where a volumetric loss of 0.42 ± 0.31 mm was recorded, whereas in the type 4 implants, the mean gain of peri-implant tissue volume occurred mainly in the apical section (0.83 ± 0.51 mm). These tissue volume values were significantly different between both groups for the whole ROI (*p* = 0.0452), as well as for the marginal region (*p* = 0.0274) (Figs. 4 and 5).

### Peri-implant health

After 6 months, 4 of 16 (25.0%) type 1 placed and 2 of 16 (12.5%) type 4 placed implants were diagnosed with peri-implant mucositis (i.e., presence of peri-implant signs of inflammation —BOP— without progressive bone loss). Otherwise, 12 type 1 implants (75.0%) as well as 14 type 4 implants (87.5%) did not show any signs of inflammation, indicative of healthy peri-implant tissues (i.e., absence of peri-implant signs of inflammation —BOP— without progressive bone loss).

After 12 months, 5 type 1 implants (31.25%) and 6 type 4 implants (37.5%) were diagnosed with peri-implant mucositis. At that point, 11 (68.75%) type 1 and 10 type 4 implants (62.5%) remained healthy.

At both follow-ups, there were no significant differences between the two groups as shown in Table 4.

**Table 4:** Diagnosis at the 6- and 12-month follow-ups, at the implant level (*n* = 32)

Diagnosis	Type 1 Implants 6 months	Type 4 Implants 6 months	Total 6 months	*p* value	Type 1 Implants 12 months	Type 4 Implants 12 months	Total 12 months	*p* value
Mucositis	4 (25.00%)	2 (12.50%)	6 (18.75%)	0.365	5 (31.25%)	6 (37.5%)	11 (34.40%)	0.710
Health	12 (75.00%)	14 (87.50%)	26 (81.25%)		11 (68.75%)	10 (62.5%)	21 (65.60%)	

*p < 0.05

### Factors potentially affecting peri-implant health

The logistic regression analysis did not find any significant association between the different variables and the peri-implant health results.

- Patient age at implantation (6 months: *p* = 0.626; 12 months: *p* = 0.984)
- OA-OT concept (6 months: *p* = 0.265; 12 months: *p* = 0.503)
- History of periodontitis (6 months: *p* = 0.733; 12 months: *p* = 0.888)
- Bone grafting (6 months: *p* = 0.737; 12 months: *p* = 0.124)

## Discussion

Immediately placed implants in the esthetic region showed relatively stable peri-implant tissue volume and comparable peri-implant health after 12 months. In fact, a cumulative total volume gain was achieved among the implants, from which the major gains corresponded to the apical area accompanied by minimal tissue loss at the marginal area. There was a 100% implant/prosthesis survival rate and no occurrence of peri-implantitis within the intervention groups. Thus, both type 1 and type 4 implant placement protocols offer similar favorable clinical results for rehabilitating the anterior maxillae.

The novelty and advantages of the three-dimensional analysis of the peri-implant tissue lie in its accuracy, easy handling, and the image superimposition possibilities that allow for linear, surface, and volumetric assessments. With the aid of this technology, subtle differences within peri-implant tissue morphology can be detected. In this sense, soft tissue contour alterations in a specifically restricted area, situated 2 to 3 mm apical to the gingival zenith, with a discrete mesiodistal distribution of 2 mm to the gingival zenith could be observed at 3, 6, and 12 months after immediate implantation and loading [10]. Indeed, dimensional analysis can be sectioned in any number of regions/volumes of interest. A selected rectangular volume of tissue located 2 mm above the mucosal margin with dimensions of 2 × 2 × 6 mm showed a significant volume loss of 17.4% when a healing abutment was placed, in comparison with an 11.9% volume loss in the group that received an immediate provisional restoration after immediate implant placement [13]. In the present study, ROI was divided into apical and marginal sections, displaying peri-implant tissue gain in the apical region and, in contrast, tissue loss at the marginal region in both intervention groups.

Complete preservation of anterior soft tissue morphology after tooth extraction is still an almost unattainable clinical scenario. Obstacles such as the need for precise surgical skills, the unpredictability of tissue remodeling dynamics and the frequent insufficiency of buccal bone compromise immediate implant therapy success [13, 14]. Certainly, physiological resorption of the underlying bundle bone is presumably the main reason behind soft tissue contour alterations [15]. Different studies have reported that the majority of both soft and hard tissue loss occurs during the first 3 months after tooth extraction [10, 16] and then tends to stabilize after 1 year [10, 14]. A relatively recent clinical trial found an occurrence of mean mid-buccal —0.32 ± 0.44 mm— and interproximal —0.17 ± 0.29 mm— recessions around immediately placed implants after 12 months [17]. These results align with our data, in which we found a marginal loss of tissue in the type 1 implant group after 12 months. Otherwise, peri-implant tissue surrounding type 1 implants located between 1 to 3 mm above the mucosal margin showed better thickness stability in comparison with the type 4 implantation group [18]. Thus, in most cases modest but clinically acceptable peri-implant tissue loss is expected after immediate implantation.

There are different factors that may affect the stability of soft peri-implant tissue after immediate implantation protocols, such as gingival phenotype and flap elevation [17, 19]. Moreover, the presence of less than 2 mm of keratinized mucosa around implant-supported restorations is associated with an increased occurrence of mucosal recessions and peri-implantitis [20–22]. Even though immediate implantation was associated with wider keratinized mucosa and no signs of progressive bone loss were detected in our study, peri-implant mucositis was present in more than 30% of implants in the type 1 implantation group, without significant differences as compared to the type 4 implantation group. However, these findings are in accordance with studies that report its incidence at between 20 and 48.5% at the implant level for immediate implants [23, 24]. Therefore, the presence of local peri-implant inflammation, which does not necessarily lead to peri-implantitis, is a common finding among both type 1 and type 4 implants, irrespective of the width of their surrounding keratinized tissue.

Bone augmentation procedures were performed around most implants (71.9% — 15 immediate and 9 delayed implants), divided between gap filling and lateral augmentation, without influencing peri-implant tissue stability or health. Accordingly, a study also found no significant differences when gap filling was performed around type 1 implants in comparison with type 4 implants preceded by ridge preservation either in terms of soft tissue width and esthetics, or marginal bone width and level [25]. Similarly, other studies found no difference regarding horizontal bone width whether grafting was or was not performed during immediate implantation with an intact buccal bone plate [26, 27]. In our study, gap filling was performed when the distance between the alveolar bone buccal plate and the implant was more than 2 mm; otherwise, lateral bone augmentation was performed when there were bone dehiscences that could compromise therapy success.

Primary and secondary implant stability were evaluated by means of periotest-assessed implant micromobility, in which values can be correlated with osseointegration levels; thus, an inferior PTV means better osseointegration/implant stability [28]. After 12 months, the PTV value of the type 1 implants became more negative on average, which could mean a better osseointegration over time. A prospective clinical study also showed that implant stability, by means of periotest, initially deteriorated during the first 3–6 months, reflecting bone remodeling around dental implants. Subsequently, the PTV values also decreased significantly after the 1-year interval [29]. However, variability between different intrinsic bone factors such as cortical thickness, trabecular bone characteristics, and even bone to implant contact area may affect the interpretation of PTV values [30]. Thus, bone remodeling around immediate implants could lead to better stability values over time, in a similar way to those achieved with delayed implants.

As with every other study, this investigation has limitations that may suggest a cautious interpretation of its results. In fact, our study includes a rather small patient collective, it has only a 12-month follow-up and does not make a proper assessment of peri-implant tissue esthetics. It also has a non-uniform distribution of bone augmentation procedures between groups, which may affect the final outcome measures. Moreover, even though intraoral scanning provides a valid assessment of the peri-implant tissue dimensions over time, it is not possible to completely differentiate whether the tissue changes are absolutely restricted to soft or hard tissue; for this, a complementary cone beam computed tomography analysis could be performed. In this context, patients would need additional radiation exposure for the procedure, which we avoided during this study by only using the intraoral scan for volumetric analysis. Furthermore, the performance of a 3D scan before tooth extraction in the case of type 4 implants and a longer follow-up could better determine the stability of soft tissue around implant-supported restorations. In this sense, an objective assessment of soft tissue stability and esthetics such as the Pink Esthetics Score complemented by patient-reported outcome measures assessing patients’ perceived esthetics could reveal whether the modest soft tissue loss during immediate implantation is clinically relevant. Apart from that, a randomized controlled trial with a longer follow-up, aiming at the same objectives of our study, could lead to less biased results and confirm the non-inferiority of immediate implantation protocols in comparison with delayed implantation protocols in terms of volumetric tissue changes.

## Conclusions

Our results suggest that type 1 implants placed in the esthetic region can suffer more tissue remodeling than type 4 implants. Although, both implant placement protocols achieve comparable peri-implant health, marginal tissue remodeling should be considered when deciding for immediate implant placement for rehabilitating the anterior maxillae.

## Data Availability

The data analyzed during the present study are available from the corresponding author on reasonable request.

## Abbreviations

ROI: Region of interest
PD: Probing depth
BOP/SUP: Bleeding/suppuration on probing
PI: Modified plaque index
KM: Keratinized mucosa
MR: Mucosal recession
PTV: Periotest value recorded
OAOT: One abutment–one time
LRG: Lateral ridge augmentation
